# Presynaptic Ionotropic Receptors Controlling and Modulating the Rules for Spike Timing-Dependent Plasticity

**DOI:** 10.1155/2011/870763

**Published:** 2011-09-15

**Authors:** Matthijs B. Verhoog, Huibert D. Mansvelder

**Affiliations:** Department of Integrative Neurophysiology, Center for Neurogenomics and Cognitive Research (CNCR), Neuroscience Campus Amsterdam, VU University Amsterdam, Room C-440, De Boelelaan 1085, 1081 HV Amsterdam, The Netherlands

## Abstract

Throughout life, activity-dependent changes in neuronal connection strength enable the brain to refine neural circuits and learn based on experience. In line with predictions made by Hebb, synapse strength can be modified depending on the millisecond timing of action potential firing (STDP). The sign of synaptic plasticity depends on the spike order of presynaptic and postsynaptic neurons. Ionotropic neurotransmitter receptors, such as NMDA receptors and nicotinic acetylcholine receptors, are intimately involved in setting the rules for synaptic strengthening and weakening. In addition, timing rules for STDP within synapses are not fixed. They can be altered by activation of ionotropic receptors located at, or close to, synapses. Here, we will highlight studies that uncovered how network actions control and modulate timing rules for STDP by activating presynaptic ionotropic receptors. Furthermore, we will discuss how interaction between different types of ionotropic receptors may create “timing” windows during which particular timing rules lead to synaptic changes.

## 1. Introduction

A central question in neuroscience is how memories are formed and stored in the brain. Studies in laboratory animals have demonstrated that learning occurs through activity-dependent synaptic strength modification [1]. Given the sequential nature of many of our memories, it may come as no surprise that the temporal order of neuronal activity is a key determinant of synaptic plasticity. The order of presynaptic and postsynaptic action potential firing within a millisecond time window leads to either strengthening or weakening of synapses [2–6]. Timing principles for synaptic plasticity also hold for human synapses [7]. 

The involvement of postsynaptic ionotropic N-methyl-D-aspartate receptors (NMDARs) in synaptic plasticity and spike-timing-dependent plasticity (STDP) has been well established [8]. Coincident pre- and postsynaptic firing is detected by postsynaptic NMDARs (post-NMDARs) that take on the role of coincidence detectors due to their multiple requirements for activation, which include the binding of glutamate, a signal of presynaptic activity, and depolarisation, a signal of postsynaptic activity. The depolarisation of the receptor is necessary in order to remove the magnesium ion (Mg^2+^) blocking the channel pore at more hyperpolarised potentials [9], and is thought to be delivered by back propagation of somatic action potentials [10]. Activated postNMDARs then permit the influx of calcium (Ca^2+^), which can set in motion intracellular Ca^2+^-dependent mechanisms that lead to transient or lasting changes in synaptic strength via changes in postsynaptic *α*-amino-3-hydroxy-5-methyl-4-isoxazole propionic acid receptor (AMPARs) expression and phosphorylation. 

Temporal rules for spike-timing-dependent plasticity (STDP) are not the same for every synapse; a diversity exists depending on brain area, neuron type, and location along dendrites [11–14]. In juvenile rodent hippocampus, the window for synaptic modification is restricted to about 40 ms [14–17] and a sharp switch of the direction of synaptic change exists at the 0 millisecond timing interval. In neocortical pyramidal neurons, the shape of the temporal STDP window depends on the location of synapses along the apical dendrite [12]. In layer (L) 5 pyramidal neurons, proximal and distal synapses exhibit a progressive distance-dependent shift in the timing requirements for the induction of long-term potentiation (LTP) and long-term depression (LTD) [18, 19]. The mechanisms underlying these differences in timing rules rely on postsynaptic Ca^2+^ dynamics induced by back propagating action potentials: synapses at proximal dendritic locations experience sharper dendritic Ca^2+^ dynamics than distal synapses due to broadening of the action potential at distal dendrites [10, 18–21]. As a result of dendritic depolarisation, more Ca^2+^ enters the postsynaptic neuron through NMDARs and voltage-gated Ca^2+^ channels (VGCCs) [10, 18]. 

In recent years, it has become clear that other factors beyond Ca^2+^ influx through postNMDARs control STDP and contribute to a diversity of timing rules at glutamatergic synapses [22, 23]. In particular presynaptic ionotropic receptors, such as NMDARs and nicotinic acetylcholine receptors (nAChRs), can determine temporal rules and the sign of plasticity in STDP. Presynaptic ionotropic receptors located on presynaptic terminals are ideally suited to influence the efficacy of synaptic transmission by directly affecting neurotransmitter release [24–26]. Short- and long-term activity-dependent modulation of the efficacy of synapses is crucial for regulating the flow of information throughout the nervous system and has been implicated in many neural processes, including learning. 

For several of the presynaptically located ionotropic glutamate receptors—AMPARs, kainate receptors (KARs) and NMDARs—it has been reported that they not only regulate neurotransmitter release, but are also involved in short- and long-term modification of synapse strength [27]. For instance, hippocampal CA3 mossy fibre synapses onto pyramidal neurons show frequency facilitation on a seconds time-scale that involves activation of presynaptic kainate autoreceptors [28]. On a minutes time scale, these presynaptic kainate receptors participate in the induction of LTP [29]. However, in these studies, the role of presynaptic kainate receptors in the temporal rules of spike-timing-dependent plasticity was not considered.

## 2. Presynaptic NMDA Receptor-Dependent Spike-Timing-Dependent Plasticity

There is an abundance of anatomical and physiological evidence for the existence of presynaptic NMDARs (pre-NMDARs) in the mammalian neocortex [30], and many noncortical areas including the striatum [31, 32], hippocampus [33–35], and cerebellum [36–38]. Physiological evidence for the existence of preNMDARs came from the observation that activation of NMDARs could lead to changes in transmitter release [39]. It is now clear that preNMDARs are not only involved in modulating transmitter release, but also have a prominent role in synaptic plasticity [30, 40]. In fact, in several cortical areas, spike-timing-dependent synaptic depression (tLTD) depends exclusively on preNMDARs and not on postNMDARs. 

The involvement of preNMDARs in STDP was first shown at synapses between connected pairs of visual cortex L5 pyramidal neurons [41]. At these synapses, a stimulation protocol where the postsynaptic neuron was brought to spike before the presynaptic neuron (“post-before-pre”) induced tLTD that was sensitive to NMDA antagonists. Both CV-analysis and the reduction in short-term depression that accompanied tLTD indicated a presynaptic expression mechanism. The authors reasoned that since pre- and postsynaptic activity was required for tLTD induction, but expression was presynaptic, a retrograde messenger would be required. A prime candidate was endocannabinoids (eCB), which are known retrograde messengers, capable of modulating presynaptic neurotransmitter release through CB1 receptors (CB1R) located on presynaptic terminals (Wilson and Nicoll [42]). tLTD was indeed found to be dependent on eCB signaling, since it was blocked by the CB1 receptor antagonist AM251. eCB release by neurons is typically triggered by an increase in intracellular Ca^2+^ concentration (DiMarzo [43, 44]). Indeed, postsynaptic Ca^2+^ chelation with intracellular BAPTA blocked the induction of tLTD. Presynaptic activity alone in presence of the CB1R agonist ACEA without postsynaptic spiking led to eCB-dependent LTD (cLTD), suggesting the requirement of postsynaptic activity for tLTD serves only to trigger the release of eCBs. 

Surprisingly, cLTD was still sensitive to bath applied NMDAR antagonists, but since cLTD was independent of postsynaptic activity, it is unlikely that the NMDARs are located postsynaptically, because these would not be activated without postsynaptic depolarization. Also, NMDAR stimulation led to an increase in mEPSC frequency, suggesting preNMDARs were located presynaptically. Based on these observations, the authors concluded that the most parsimonious explanation was that NMDARs involved in tLTD are located presynaptically. 

More reports on preNMDAR-dependent tLTD in visual cortex [45] and somatosensory cortex [46] soon followed. There, tLTD was also shown to be sensitive to bath applied NMDAR antagonists, but to be independent of postNMDARs, since tLTD persisted when postNMDARs were blocked by loading postsynaptic neurons with the use-dependent NMDAR blocker MK-801 [45, 46] or by hyperpolarizing the postsynaptic neuron at the time of the presynaptic spike [46]. The non-postsynaptic NMDARs were assumed to be located presynaptically from the observed effect of NMDAR stimulation on the frequency of spontaneous excitatory postsynaptic currents (EPSCs) [45] and the amplitude of evoked AMPAR-mediated EPSCs [46], or by immunohistochemistry [45]. 

The definite proof that NMDARs involved in tLTD were indeed located on presynaptic neurons came from an elegant study in the rodent barrel cortex [23], where STDP plays a role in sensory whisker map formation [47]. In L4 to L2/3 synapses, a pre-before-post induction protocol induced timing-dependent LTP (tLTP), and the reverse (post-before-pre) induced timing-dependent LTD. Rodriguez-Moreno and Paulsen [23] demonstrated that postsynaptic MK-801 blocked tLTP, but not tLTD whereas presynaptic MK-801 blocked tLTD, but not tLTP. These results showed that tLTP and tLTD are dependent on different NMDARs, namely postNMDARs and preNMDARs, respectively. 

It is important to note that most of the examples of tLTD reported above are assumed to be mediated by NMDARs located on, or at least close to, the synaptic terminals, because of the observed effects of NMDAR stimulation on transmitter release [41, 45, 46]. The reasoning behind this is that the increase in intracellular Ca^2+^ following NMDAR activation is spatially limited to micro- or nanodomains, so in order for NMDAR activation to affect the Ca^2+^ sensitive transmitter release processes [48], these receptors must lie close to the synaptic terminal. The legitimacy of this assumption has been questioned, however, by the recent finding that subthreshold depolarization following activation of somatodendritic NMDARs can affect axonal Ca^2+^ levels through recruitment of VGCCs [49]. Moreover, a follow-up study failed to detect changes in axonal Ca^2+^ levels when directly applying NMDA to axonal compartments of visual cortex L5 pyramidal neurons [50]. These new insights call for some caution when interpreting NMDAR-mediated effects on synaptic transmission. Therefore, although it remains difficult to imagine how such somatodendritic NMDARs on presynaptic neurons would be recruited by tLTD induction paradigms used in the above studies, their involvement cannot be excluded. 

To date, all forms of cortical preNMDAR-dependent STDP reported in the literature involve tLTD [23, 41, 45, 46, 51], so it is unknown whether these presynaptic receptors can also mediate tLTP. However, Duguid and Smart reported an intermediate form of LTP of inhibition in basket and stellate cell synapses onto Purkinje cells in the cerebellum; pairing presynaptic spiking with postsynaptic depolarisation resulted in a short period (2-3 min) of depolarisation-induced suppression of inhibition (DSI), which was followed by a prolonged period (up to 15 minutes) of “depolarisation-induced potentiation of inhibition” (DPI) [37]. DPI has similarities with forms of preNMDAR-dependent plasticity mentioned above. Firstly, DPI induction also requires correlated pre- and postsynaptic activity. Secondly, DPI relies on preNMDARs since it is abolished by AP-5, but postsynaptic Purkinje cells do not express NMDARs at this age [52]. In addition, NMDAR subunits colocalised with GAD65/67 and synaptophysin, strongly suggesting that NMDARs are located at the presynaptic terminal. These results show that synaptic activity- and preNMDAR-dependent plasticity can also be involved in potentiating synapses [37]. 

Having NMDARs at presynaptic terminals involved in STDP raises questions on the nature of the underlying induction and expression mechanisms; firstly, how do preNMDARs become activated? Secondly, how does preNMDAR activation lead to a lasting change in synaptic efficacy? And thirdly, where is the change expressed? In all the examples mentioned above, tLTD was accompanied by changes in short-term plasticity. This most likely reflects changes in release probability, pointing to a presynaptic site of expression. It is not unlikely that it is the presynaptic influx of Ca^2+^ through activated preNMDARs that triggers the lasting change in release probability. To date, the precise mechanisms by which such an NMDAR-mediated Ca^2+^ influx can induce such changes have not been directly investigated, so the answer to the second question remains elusive. 

**Figure 1:**
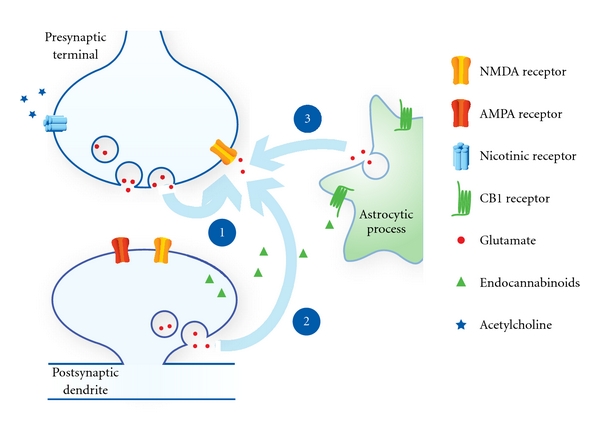
Three possible sources of glutamate for preNMDAR activation. (1) The first and most straightforward route would be that preNMDARs are auto-receptors that receive glutamate from the same terminals on which they are located. A problem with this scenario is that the necessary depolarisation for NMDAR activation may have ended by the time glutamate has reached the receptor. Therefore, preNMDARs will either need to be less voltage-sensitive or require some other source of depolarisation. (2) A second possibility is that glutamate derives from the *post*synaptic cell. In a post-before-pre pairing protocol, the depolarisation of the postsynaptic neuron can elicit glutamate release which will activate preNMDARs when these are depolarised by the presynaptic action potential. (3) eCBs, released postsynaptically following depolarisation, can act on CB1Rs on nearby astrocytes to induce astrocytic glutamate release. The question is whether this mode of glutamate delivery will be fast enough to play a role in the tLTD induced at small pairing intervals in the range of a few tens of milliseconds.

How are preNMDARs activated? As mentioned before, NMDARs require both depolarisation and binding of glutamate to become activated. Presynaptic action potential firing provides an obvious source of depolarisation to preNMDARs, but the source of glutamate acting on these receptors is less obvious. A number of possible sources can be identified (Figure 1). Firstly, as other presynaptic receptors, preNMDARs can be activated by neurotransmitter released from the same nerve terminals on which the receptors themselves are located, thereby acting as autoreceptors [24, 26, 39]. Alternatively, glutamate may be released postsynaptically and act as a retrograde signal to activate preNMDARs. Finally, glutamate can derive from sources outside the synapse, such as spill-over from synapses in the vicinity or glutamate release from nearby astrocytic processes. 

At first glance, a role for preNMDARs as auto-receptors on glutamatergic terminals may seem unlikely, because by the time glutamate released from the terminal on which the receptors are located has reached the preNMDARs, the depolarisation causing its release may already have ended. Thereby, Mg^2+^ would not leave the channel once glutamate reaches the receptor. However, the preNMDARs on which tLTD of mouse barrel cortex L4 to L2/3 synapses depends were shown to contain NR2C/D subunits [51], which are known to be less voltage-sensitive [53]. Therefore, they may be well-suited as preNMDARs in this form of tLTD, being able to activate when glutamate binds even without a strong depolarisation. But tLTD does not always rely on less voltage-sensitive NMDARs; preNMDAR-dependent tLTD at rat L5 to L5 visual cortex pyramidal neuron synapses and mouse L2/3 horizontal connections in barrel cortex relied on NR2B subunit-containing NMDARs, which tend to have a higher voltage dependency [53]. Since NMDARs are heteromeric structures, it remains possible that other NMDAR subunits coassemble with NR2B to make the receptor less voltage sensitive. If preNMDAR-dependent tLTD relies on NMDARs with low voltage-sensitivity, glutamate binding with only a mild depolarisation could be sufficient for channel opening and preNMDARs could function as auto-receptors after all. 

PreNMDARs could also be activated by postsynaptically released glutamate, which could ensure that NMDARs are glutamate bound at the time of the presynaptic action potential [54]. This was shown to be the case in DPI of interneuron to Purkinje cell synapses [37]. Since these synapses are GABAergic, preNMDARs will not act as auto-receptors. By pharmacologically blocking EAAT-mediated glutamate reuptake, the hypothesis was tested that retrograde postsynaptic release of glutamate could activate preNMDARs. Consistent with this hypothesis, subthreshold short postsynaptic depolarisation induced DPI when combined with presynaptic spiking. Thus, the authors concluded that postsynaptically released glutamate may be responsible for activating preNMDARs in this form of plasticity. Although dendritic glutamate release has been reported in cortical pyramidal neurons as well [55], it has thus far not been investigated whether preNMDAR-dependent tLTD also relies on retrograde glutamate signalling. 

The source of glutamate may also lie outside the synapse. Spill-over from neighbouring glutamatergic synapses has been suggested before as a source of glutamate in other forms of preNMDAR-dependent plasticity [56, 57]. However, in these studies neighbouring glutamatergic synapses were explicitly stimulated during plasticity induction. As a consequence, tLTD at a specific synapse with preNMDARs would then only occur if neighbouring glutamatergic synapses would be active. 

Alternatively, a potential source of glutamate may be astrocytes. In recent years, it has become clear that glial cells are intimately involved in the active control of neuronal activity, synaptic transmission, and plasticity [58]. This has led to the concept of the tripartite synapse [58–60], where communication is not limited to the pre- and postsynaptic neuronal elements, but where there is also a bidirectional communication between neurons and the astrocytes ensheathing the synapse. The potential importance of such astrocyte-neuron communication for synaptic plasticity was demonstrated recently in a study showing that astrocytic release of the neuromodulator D-serine was required for LTP at Schaffer collateral synapses onto CA1 pyramidal neurons [61], although this is not without dispute [62]. It is not unthinkable that astrocytes fulfill a similar role in preNMDAR-dependent tLTD by releasing glutamate. In fact, astrocytes have been reported to have the necessary intracellular machinery at their disposal for regulated exocytosis of glutamate [63] and such astrocyte-derived glutamate can readily activate preNMDARs [33]. Interestingly, preNMDARs have been observed in extrasynaptic regions of presynaptic terminals closely apposed to glutamate-containing synaptic-like microvesicles in astrocytic processes [33]. 

How is glutamate release triggered from astrocytes? Astrocytes express CB1 receptors which upon stimulation can trigger increases in intracellular Ca^2+^ levels leading to glutamate release [64, 65]. Therefore, postsynaptically released eCBs may deliver signals of postsynaptic activity to nearby astrocytic processes. Indeed, postsynaptically released eCBs have been shown to potentiate synapses in hippocampus by inducing glutamate release from astrocytes which in turn activated presynaptic metabotropic glutamate receptors [65, 66]. Since preNMDAR-dependent tLTD at rat L5 to L5 visual cortex synapses [41], rat L4 to L2/3 barrel cortex synapses [46], and mouse L2/3 to L2/3 barrel cortex synapses [51], depended on eCB signalling as well, eCB signalling may be a general mechanism in preNMDAR-dependent plasticity, serving to elicit glutamate release from astrocytes. 

The sequence of events that would have to take place in the case of eCB- and preNMDAR-dependent tLTD would be as follows; during post-before-pre activity the postsynaptic neuron spikes first, allowing an increase in postsynaptic intracellular Ca^2+^ levels, which induces postsynaptic eCB release. Activation of astrocytic eCB receptors induces increases in intracellular Ca^2+^ levels of the astrocyte which leads to the release of glutamate that binds to preNMDARs. The depolarisation associated with following presynaptic action potentials then activates preNMDARs and the subsequent influx of Ca^2+^ triggers some as yet unknown intracellular mechanism that leads to a persistent reduction of glutamate release. This scenario has one obvious difficulty; the fact that preNMDAR-dependent tLTD can be induced using pre-before-post pairing intervals of only a few milliseconds puts severe time constraints on all the steps necessary within such a model. This issue can potentially be resolved by considering the time-course of astrocytic Ca^2+^ signals, which typically take place on a seconds timescale [67–70]. Therefore, eCB-mediated Ca^2+^ signals in astrocytes induced by the first pairings in the plasticity induction protocol may ensure glutamate levels are elevated during subsequent pairings. Definite proof of this sequence of events from postsynaptic eCB release to preNMDAR activation by astrocytic glutamate release awaits experimental testing.

Recently, Banerjee et al. [51] reported that in *mouse* barrel cortex L4 to L2/3 synapses, preNMDAR-dependent tLTD was eCB independent. These results raise the question of what other signalling mechanisms could be at play here. One candidate molecule would be nitric oxide (NO), which has been shown to play a role in preNMDAR-dependent cerebellar LTD [36]. In fact, NO has been implicated in mediating the presynaptic component of tLTP at the same barrel cortex L4 to L2/3 synapses in mice [71]. NO derived from the postsynaptic neuron where it was released in response to postsynaptic depolarisation. Application of an NO donor resulted in an increase in miniature EPSC frequency, indicating a presynaptic action and suggesting that NO is indeed employed as a retrograde messenger at these synapses. Since NO has also been shown capable of eliciting vesicular glutamate release by astrocytes [72], it is possible that preNMDAR-dependent tLTD in the mouse brain occurs through recruitment of astrocytes by NO signalling. 

One final issue to discuss here is the frequency dependence of tLTD. Barrel cortex tLTD of L4 to L2/3 synapses [46] and tLTD of visual cortex L5 to L5 synapses [41] are two cases of preNMDAR-dependent plasticity that share many similarities; both require specifically timed pre- and postsynaptic activity, both are expressed presynaptically, and both require activation of both CB1Rs and preNMDARs. However, some differences seem to exist. Most importantly, as pointed out by Duguid and Sjöström [54], in the presence of CB1 agonists, cLTD could be induced in barrel cortex L4 to L2/3 synapses by trains of presynaptic stimulations delivered at either high (30 Hz) or low (0.1 Hz) frequencies [46]. This was not the case in L5 visual cortex neurons, where cLTD was only induced at stimulation frequencies higher than 15 Hz [41]. The latter finding is intriguing, because *t*LTD at this synapse *can *be induced at low (0.1 Hz) post-before-pre pairing frequencies. This suggests that at lower stimulation frequencies, some additional mechanism is needed besides eCB signalling. Possibly, as proposed by Duguid and Sjöström [54], tLTD at low stimulation frequencies relies on an additional retrograde signal from the postsynaptic cell. As yet, the nature of this additional messenger can only be guessed at, but perhaps investigating the involvement of NO would be a good place to start.

Together, these results indicate that preNMDARs often require the involvement of other signalling molecules or messenger systems to fulfill their role in plasticity. It is important to know what precisely leads to preNMDAR activation during STDP induction, as it has computational consequences for the role of preNMDAR-dependent tLTD in information processing. PreNMDARs functioning as auto-receptors would mean they are detectors of specific intrinsic activities of the synapse. However, if preNMDARs are activated by glutamate from neighbouring cells, preNMDAR-dependent tLTD would be not only a reflection of coinciding pre- and postsynaptic activity, but also of coinciding activity of neurons and possibly astrocytes in the surrounding network.

## 3. Modulation of Timing-Dependent Plasticity by Presynaptic Nicotinic Acetylcholine Receptors

Acetylcholine (ACh) is one of the major neurotransmitters in the brain involved in regulating neuronal network activity. The effects of ACh are mediated by two types of receptors; the metabotropic muscarinic receptors (mAChRs) and the ionotropic nAChRs. nAChRs are ion channels which open upon the binding of ACh, permitting the influx of multiple ionic species, most notably sodium and calcium, resulting in membrane depolarisation. Brain nAChRs are composed of multiple subunits, either heteromeric combinations of *α*(2–10) and *β*(2–4) subunits or homopentamers consisting of *α*7 subunits. The precise subunit composition has a profound effect on the biophysical (Ca^2+^ permeability, kinetics) and pharmacological properties (affinity, desensitization) of the receptor [73, 74]. These receptors are present throughout the brain, and are often found at somatodendritic locations, where they influence the excitability of the cell. However, just as NMDARs, nAChRs can also be found at presynaptic terminals in several brain regions, where they directly modulate excitatory glutamatergic transmission [75–81]. Most of these presynaptic nAChRs contain *α*7 subunits [77] and are thereby highly Ca^2+^ permeable [82], ideally suited to modulate the release of synaptic vesicles.

Activation of presynaptic nAChRs can induce synaptic plasticity [78]. In the ventral tegmental area (VTA) of the mesolimbic dopamine system, which is involved in reward processing, glutamatergic synapses on dopaminergic neurons can undergo LTP when presynaptic activation is paired with postsynaptic activation, similar to cortical glutamatergic synapses [78, 83]. Stimulation of presynaptic nAChRs on these synapses by nicotine also induced LTP when this activation coincided with postsynaptic activity [78]. The amount of LTP that was induced correlated with the level of increase in excitatory synaptic transmission induced by nAChR activation. These effects on synaptic transmission were insensitive to TTX, indicating that the nAChRs involved are located on, or close to the presynaptic terminals. Both changes in excitatory synaptic transmission and nicotine-induced LTP were mediated by *α*7 subunit-containing nAChRs. Nicotine-induced LTP of glutamatergic inputs to DA neurons depended on NMDAR activation, which required postsynaptic depolarisation to remove the Mg^2+^ blockade. This depolarisation could be provided by the postsynaptic nAChRs on the dopamine neurons. It was recently shown that pre- as well as postsynaptic nAChRs in the VTA are involved in increasing glutamatergic synapse function, and initiating glutamatergic synaptic plasticity [84], which may be an important, early neuronal adaptation in nicotine reward and reinforcement.

nAChRs can also modulate the rules for STDP, from locations further upstream than the presynaptic terminal [22]. In L5 pyramidal neurons of mouse medial prefrontal cortex (mPFC), pairing presynaptic and postsynaptic activity at 5 ms intervals induced a long-term strengthening of glutamatergic inputs [22]. When nAChRs were stimulated with nicotine, tLTP was eliminated and a depression of the excitatory inputs was observed. This nicotinic modulation of plasticity was abolished by inhibitors of GABA type A (GABA_A_) receptors, indicating the effects of nicotine were due to its actions on presynaptic interneurons. Different types of GABAergic interneurons found in the PFC L5 express nAChRs on their somas that activate these neurons when nicotine is present. Thereby, nAChR stimulation enhanced GABAergic inputs to L5 pyramidal neuron dendrites, resulting in reduced Ca^2+^ entry during action potential back-propagation from the soma [22, 85]. Increasing dendritic action potential propagation by burst-like stimulation of the pyramidal neuron in the presence of nicotine could restore postsynaptic Ca^2+^ to levels comparable to those seen in the absence of nicotine, and restored STDP as well, indicating that strong postsynaptic stimulation could overcome the nicotinic modulation. Thus, activation of nAChRs expressed by mPFC interneurons that inhibit dendrites can alter the rules for induction of STDP. 

In mouse hippocampus, timing-dependent plasticity can be modulated through a similar recruitment of inhibition by nAChRs on presynaptic interneurons [86]. nAChR activity could bidirectionally modulate plasticity, and the sign of synaptic change was critically dependent on the timing and localisation of nAChR activation. In CA1 pyramidal neurons, pairing high-frequency stimulation (HFS) of Schaffer collaterals with postsynaptic depolarisations resulted in short-term potentiation (STP) of these synapses [86]. With mAChRs blocked by atropine, a puff of ACh in dendritic regions of the cell during plasticity induction boosted STP into LTP [86]. This effect was attributed to stimulating postsynaptic *α*7 subunit-containing nAChRs. If, however, the ACh puff was aimed at a neighbouring connected interneuron, the same protocol could no longer induce STP. Moreover, stimulating nAChRs on nearby interneurons during a stronger plasticity induction protocol, capable of inducing LTP in control conditions, converted LTP into STP [86]. This demonstrates that timing and localization of nAChR activity in the hippocampus can determine whether LTP will occur or not. Although the authors did not further investigate the mechanisms underlying the blockade of plasticity by interneuronal nAChR activation, it is tempting to speculate that the resulting increase in inhibitory input reduces postsynaptic Ca^2+^ signals in CA1 pyramidal neurons in a similar manner as it does in L5 neurons of the mPFC [22]. Plasticity induction by HFS does not involve back propagating action potentials, but increased inhibition may reduce the activation of postsynaptic voltage-dependent channels such as NMDARs and VGCCs that would otherwise be activated and promote synaptic potentiation. 

Synaptic plasticity is critically important for cognitive function. Synaptic plasticity in the hippocampus has been associated with memory formation and synaptic plasticity in the PFC has been directly associated with attention and working memory [87]. Activation of nAChRs alters processes of synaptic plasticity in cortical and hippocampal neuronal networks. By altering Ca^2+^ dynamics during active dendritic signalling in apical dendrites, nAChRs may affect communication between cell body and distal synapses. This potentially could affect information processing in cortical neuronal networks. Alternatively, nAChRs may provide neuronal networks with the option to locally modulate synaptic plasticity, allowing a particular neuron or a particular synapse to respond differently than the average of the surrounding circuitry [86]. 

By what sources of ACh are presynaptic nAChRs activated? Endogenous cholinergic signals occur at multiple timescales, ranging from seconds to minutes [88]. Anatomical evidence shows that in rodent and human neocortex cholinergic nerve terminals establish classical synapses with closely apposed presynaptic and postsynaptic structures [89, 90], but direct physiological evidence for functional cholinergic synaptic transmission in the neocortex is lacking. In hippocampus, fast synaptic currents mediated by cholinergic transmission and *α*7 subunit-containing nAChRs have been observed in interneurons, but not pyramidal neurons [91]. Slow, tonic modes of ACh release may act on neurons in a diffuse manner, although ACh is rapidly broken down by the substantial levels of acetylcholinesterase in the neocortex [92]. Whether rapid phasic ACh changes act directly or in a diffuse manner is not known. Recently it was shown that in the interpeduncular nucleus high-frequency (20–50 Hz) stimulation of ACh neurons eventually generates postsynaptic nAChR-mediated responses via volume transmission [93, 94]. Regardless, the findings above suggest that during fast or slow ACh signalling the rules for STDP may be altered for shorter or longer time.

## 4. Potential Interplay between Presynaptic Ionotropic Receptors in STDP

Synapses can express multiple presynaptic ionotropic receptors that affect synaptic function and different types of ionotropic receptors can interact at the presynaptic level. For instance, activation of presynaptic ionotropic purinergic P2X receptors potentiates glutamate release due to the activation of *α*7-containing nAChRs coexisting on rat neocortex glutamatergic terminals [95]. Considering the involvement of preNMDARs and presynaptic nAChRs in STDP, it would be interesting to examine whether these two species of receptors may also be found at the same synaptic terminals and if so, whether a similar interplay between nAChRs and NMDARs may occur. Direct evidence for coexpression of presynaptic nAChRs and NMDARs is to our knowledge limited to one study on rat primary cortical cultures. There, axonal *α*7 nAChRs were found to modulate preNMDAR expression, implicating presynaptic *α*7 nAChR/NMDAR interactions in synaptic development and plasticity [96].

Evidence for co-expression of these receptors in postnatal animals is indirect. Firstly, in rat striatum, corticostriatal glutamate projections contain presynaptic *α*7 subunit-containing nAChRs that upon stimulation elicit glutamate release [97]. Through microdialysis studies it was shown that NMDARs could enhance glutamate release as well in this area, which the authors suggested was due to activation of preNMDARs on cortico-striatal nerve endings [31]. Secondly, in rat hippocampus, presynaptic *α*7 subunit-containing nAChRs have been reported to exist on excitatory presynaptic terminals [98], where they increase spontaneous and evoked glutamate release [99]. These could well be the same synapses as where transmitter release-modulating preNMDARs have been reported on a number of occasions [33–35]. Finally, in the neocortex where the preNMDAR-dependent forms of tLTD described above were observed, presynaptic nAChRs have also been reported [100]. Thus, several candidate synapses exist for co-expression of presynaptic NMDARs and nAChRs. 

Co-expression of these presynaptic ionotropic receptors could have several distinct, though not mutually exclusive, consequences for STDP. Firstly, since presynaptic nAChRs promote LTP, but preNMDARs control LTD, there is the potential for an exciting competition to take place between potentiation and depression mechanisms at the presynaptic terminal. It must be noted, however, that all examples given of presynaptic nAChRs promoting LTP are non-cortical (hippocampus, VTA) and LTD promoting preNMDARS are cortical. Secondly, a synergistic interplay could take place. The most notable similarity between nAChRs and NMDARs is that they are both permeable to Ca^2+^. In fact, upon activation, *α*7 subunit-containing nAChRs permit a Ca^2+^ influx that rivals that of NMDARs [82]. The important difference with NMDARs is, however, that nAChRs do not have the voltage-dependent Mg^2+^ block. So, activation of nAChRs at resting membrane potentials directly leads to Ca^2+^ influx without the need for depolarisation. At depolarized potentials (>0 mV), however, an Mg^2+^-dependent inward rectification takes place at nAChRs that restricts the flow of current to very low levels [82, 101]. In that sense, activity of nAChRs and NMDARs may complement each other, acting at more or less distinct ranges of membrane potentials. 

Thirdly, a direct interaction by which the activity of one receptor affects the other may exist. If NMDARs and nAChRs are expressed at the same synaptic terminal, local intracellular Mg^2+^ levels may lead to direct interaction between nAChRs and NMDARs; activation of NMDARs can result in a substantial increase in the intracellular concentrations of free Mg^2+^ [102]. This particularly affects *α*7 subunit-containing nAChRs, which have stronger Mg^2+^-dependent inward rectification than *β*2 subunit-containing nAChRs [101]. Therefore, at depolarized potentials, the increased Mg^2+^ levels following NMDAR activation can act to inhibit nAChRs and limit further Ca^2+^ influx through *α*7 subunit-containing nAChRs. This crosstalk may represent a means by which rapid rise in intracellular Ca^2+^ concentrations via activation of NMDARs and nAChRs can be tightly controlled, so that intracellular Ca^2+^ overloading is avoided [103]. Such control over Ca^2+^ signals may be very important for plasticity processes and indeed, a coregulation of postsynaptic intracellular Ca^2+^ levels by NMDARs and *α*7-containing nAChRs to control synaptic plasticity has been proposed [104]. 

The inverse, nAChRs affecting the activity of NMDARs, is also possible, albeit indirectly via intracellular signalling pathways. It has been shown that *α*7-containing nAChRs can activate calcineurin (PP2B), a Ca^2+^-sensitive enzyme, that when activated can lead to a reduction of the NMDAR-mediated current decay time [105]. By controlling the activity of PP2B, nAChRs can regulate NMDAR transmission and synaptic plasticity [103, 105, 106]. Also, Ca^2+^ signals initiated by somatic or postsynaptic nAChRs have been found to specifically reduce the amplitude of postNMDAR-mediated currents through a Ca^2+^-calmodulin-dependent process [107]. Having two routes through different ionotropic receptors towards plasticity modulation could endow the synapse with the ability to have different learning rules for different modes of processing, for example, in the presence or absence of ACh.

## 5. Conclusion

Presynaptic ionotropic receptors control and modulate activity-dependent synaptic plasticity. Activation of these presynaptic receptors provides synapses with flexibility in the temporal rules for synaptic strengthening and weakening. Thereby, the presence or absence of specific neurotransmitters can create windows during which specific timing of neuronal activity will lead to synaptic changes or not. For instance, Hebbian plasticity is enhanced by behavioral relevance and attention, particularly in adults. Attentional gating of plasticity may be provided by neuromodulators such as ACh released in cortex by basal forebrain inputs. In addition, in barrel cortex, whisker map plasticity in S1 and other areas requires ACh, and pairing of whisker stimuli with ACh application drives receptive field plasticity [108]. This suggests that presynaptic ionotropic receptors may fundamentally gate or modify Hebbian learning rules during appropriate behavioral contexts. It will be interesting to learn from future research whether other types of presynaptic ionotropic receptors besides NMDARs and nAChRs are involved in controlling and shaping the rules for STDP.
